# JSME: a free molecule editor in JavaScript

**DOI:** 10.1186/1758-2946-5-24

**Published:** 2013-05-21

**Authors:** Bruno Bienfait, Peter Ertl

**Affiliations:** 1 Molecular Networks GmbH, Henkestrasse 91, D-91052 Erlangen, Germany; 2 Novartis Institutes for BioMedical Research, Novartis Campus, CH-4056, Basel, Switzerland

## Abstract

**Background:**

A molecule editor, i.e. a program facilitating graphical input and interactive editing of molecules, is an indispensable part of every cheminformatics or molecular processing system. Today, when a web browser has become the universal scientific user interface, a tool to edit molecules directly within the web browser is essential. One of the most popular tools for molecular structure input on the web is the JME applet. Since its release nearly 15 years ago, however the web environment has changed and Java applets are facing increasing implementation hurdles due to their maintenance and support requirements, as well as security issues. This prompted us to update the JME editor and port it to a modern Internet programming language - JavaScript.

**Summary:**

The actual molecule editing Java code of the JME editor was translated into JavaScript with help of the Google Web Toolkit compiler and a custom library that emulates a subset of the GUI features of the Java runtime environment. In this process, the editor was enhanced by additional functionalities including a substituent menu, copy/paste, drag and drop and undo/redo capabilities and an integrated help. In addition to desktop computers, the editor supports molecule editing on touch devices, including iPhone, iPad and Android phones and tablets. In analogy to JME the new editor is named JSME. This new molecule editor is compact, easy to use and easy to incorporate into web pages.

**Conclusions:**

A free molecule editor written in JavaScript was developed and is released under the terms of permissive BSD license. The editor is compatible with JME, has practically the same user interface as well as the web application programming interface. The JSME editor is available for download from the project web page http://peter-ertl.com/jsme/

## Background

A program for the input and editing of molecules is an indispensable part of every cheminformatics or molecular processing system. Such a program is known as a molecule editor, molecular editor or structure sketcher. Its function is to facilitate entry of molecules or reactions into a computer with help of mouse and keyboard actions, and recently also by using a touch screen. Molecule editors are used to create chemical illustrations or as tools to draw queries when searching chemical databases or entering molecules when calculating various molecular properties.

One of the most important areas where the interactive molecule structure input is needed is molecule editing within web browsers. The World Wide Web, introduced originally as a medium for the exchange of scientific information, is affecting in meantime practically all aspects of our life, but scientific and technical applications still benefit proportionally more from the web technology. Scientific computing is moving more and more in the direction of web services and cloud computing, with servers scattered all around the Internet, and the web browser is becoming the universal scientific user interface. Chemistry is no exception from this trend and input of molecular structures directly within a web browser is therefore of utmost importance.

Some time ago one of the authors of this article reviewed various techniques for entering chemical structures on the web [1]. At that time the web-based molecule editing was clearly dominated by Java applets, with nearly 20 such programs listed in the review. This situation, however, is changing fast. This change of paradigm is caused by several factors. One is an explosive advent of handheld devices, like tablets and smartphones. These devices use touch screen as a user interface and generally do not support Java applets. Other reasons for the demise of molecular editing applets (and applets in general) are increasing requirements on their maintenance. Vast possible combinations of Java versions, plugins, browsers and operating systems make support of applets very demanding. The maintenance burden, as well as arising Java security issues led to situation that several companies, as well as public service providers, completely banned Java applets from their web sites. It is clear that the web-based cheminformatics community needs an alternative to Java applets. The solution seems to be JavaScript.

JavaScript is an interpreted programming language living in web browsers and, despite its name, has no direct relation to Java. For many years, JavaScript has been used only as a tool to add simple interactivity to web pages. Thanks to the effort of several companies spearheaded by Google, including development of fast JavaScript engines and several powerful libraries, that hide differences in JavaScript implementations between various browsers, JavaScript gained momentum and became one of the major components of the so called Web 2.0 paradigm. JavaScript together with advanced HTML5 technology supports the development of complex and powerful applications running within a web browser. As a result, various cheminformatics applications written in JavaScript are appearing, including several tools of different level of complexity and user friendliness supporting drawing of molecules [2-9]. Another example of successful application of JavaScript in cheminformatics is conversion of Jmol, a popular 3D molecule viewer from Java into pure JavaScript-powered JSmol [10].

The situation discussed above prompted the authors to port the popular JME Java applet to JavaScript and release it as a free tool to the Internet cheminformatics community. In analogy to the JME name we decided to call the new editor JSME.

## Implementation

### JSME molecule editor development

**Figure 1:**
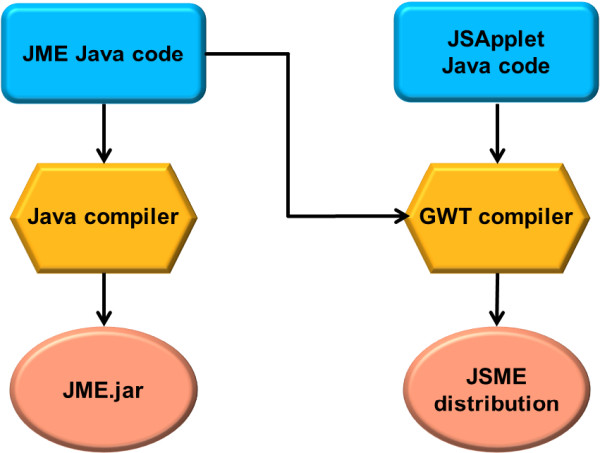
Overview of the process of the conversion of the JME Java source code into a Java applet and the JSME JavaScript distribution.

In this article, a JSME molecule editor written in JavaScript is described. JSME is a direct successor of the JME editor, a Java applet for drawing, editing, and displaying molecules and reactions directly within a web page [11]. The editor was originally written by one of the authors of this article at Comenius University in Bratislava in QuickBASIC. The program was later translated into Java (and named JME on this occasion) to be used as a structure input tool for the in-house web-based cheminformatics system at Ciba-Geigy and later at Novartis [12]. Due to many requests, the JME editor was released in 1998 to the public and is currently probably still the most popular molecular entry system on the web. Users value on JME mostly its simple and intuitive user interface and small size (only 38 Kbytes) enabling fast loading. But the changing situation in the web discussed in the previous section prompted us to look for an alternative to Java. At the same time, however, it would be of advantage to base the development of a new molecule editor on the JME code, incorporating in this way the experience and feedback of thousands of JME users who helped to make the code well validated and stable. The answer to this challenge was conversion of the JME Java code to JavaScript. To perform this task, we used the Google Web Toolkit (GWT) [13] and a custom JSApplet library. The whole procedure is schematically shown in Figure 1.

The GWT features a compiler that compiles Java code into heavily optimized JavaScript. GWT was developed by Google to support creation of rich Internet applications using the Java programming language, taking in this way advantage of many experienced Java programmers and numerous development tools, but without the necessity to deploy the created application as Java applets. GWT handles the differences in JavaScript implementations between various web browsers by producing the code that is specific for each browser. Google provides also plugins for the Eclipse [14] integrated development environment for code editing, browsing and refactoring. A very useful feature of the GWT Eclipse plugin is support for easy debugging of the Java code running inside a web browser. GWT comes with a library that emulates a small subset of the Java Runtime Environment (JRE). This subset, however, does not include the Java Abstract Window Toolkit (AWT) that is needed to compile the JME Java code into JavaScript. The Java AWT is a library that provides graphical user interface elements like windows, buttons or menus, event management (mouse or keyboard interaction), layout system, 2D graphics or data transfer with the system clipboard. Porting the whole library to GWT would be a very demanding task. Fortunately, JME is a relatively old Java program written in Java version 1.0 without any sophisticated Java GUI features that would require the Swing toolkit (the Swing graphics is more recent and considerably more complex than AWT). To support the conversion we implemented the JSApplet (JSA) library that provides the subset of AWT functionalities that are needed by JME. In JME the AWT java class Graphics is used to depict the molecular structures. The JSA implementation of this class maps the primitive graphics command (e.g. drawLine, drawRectangle, …) to the Scalar Vector Graphics (SVG) engine that is available in most web browsers, except Internet Explorer version 6, 7 and 8 where the Vector Markup Language (VML) is used instead. The AWT layout system was implemented using the Apache Harmony library from the Apache Foundation [15]. The AWT widgets buttons, labels, text fields and multiple choice menu were mapped one to one to browser corresponding native widgets. The AWT Frame and popup menu were implemented with the help of the GWT Mosaic library [16]. The GWT browser independent events were used to create the AWT events.

### JSME installation

When adding the JSME editor into a HTML page one has to provide a placeholder for the editor in the document (typically a HTML DIV element), include a bootstrap JavaScript file and define a JavaScript function to initialize the JSME. The JSME distribution itself consists of already mentioned bootstrap JavaScript file and six HTML files. Each HTML file contains highly optimized JavaScript code for one or several specific browsers. The main purpose of the bootstrap file is to detect the browser type, select the matching HTML file and load it. The JSME distribution package contains also a HTML page (JSME.html) with an example editor installation. The appearance of the JSME editor may be modified by calling the options() function with respective parameters. In this way the web developer can choose between the “classical” and new editor look, determine whether query or reaction input should be enabled, how the stereochemistry should be handled, whether nitro or related groups should appear in polar form or with symmetrical pentavalent nitrogen atoms (often a source of serious disagreement between cheminformatics experts) and so on. The full list of keywords is available at the JSME Homepage. These options are the same as used in the JME editor, therefore update from JME to JSME should be easy.

The size of the JSME code varies between 257 and 271 Kbytes according to the browser. The Java code that is common to JSME and JME accounts for about 31% of the JavaScript code generated by GWT. The size of the compiled JME used as a basis for JSME translation is 55 Kbytes, slightly larger than that of stand-alone JME, since this code contains several additional features. In practice the actual download size of JSME is about 110 Kbytes because most web servers provides on the fly compression of the downloaded files. Unlike JavaScript files, the download size of the Java applet jar file (a compressed archive file) cannot be reduced by the web server. Thus the JSME download size is only about two times larger than that of JME itself. In practice, the JSME is ready to be used even sooner than the JME, because the applets are delayed by the Java Runtime Environment initialization phase.

**Figure 2:**
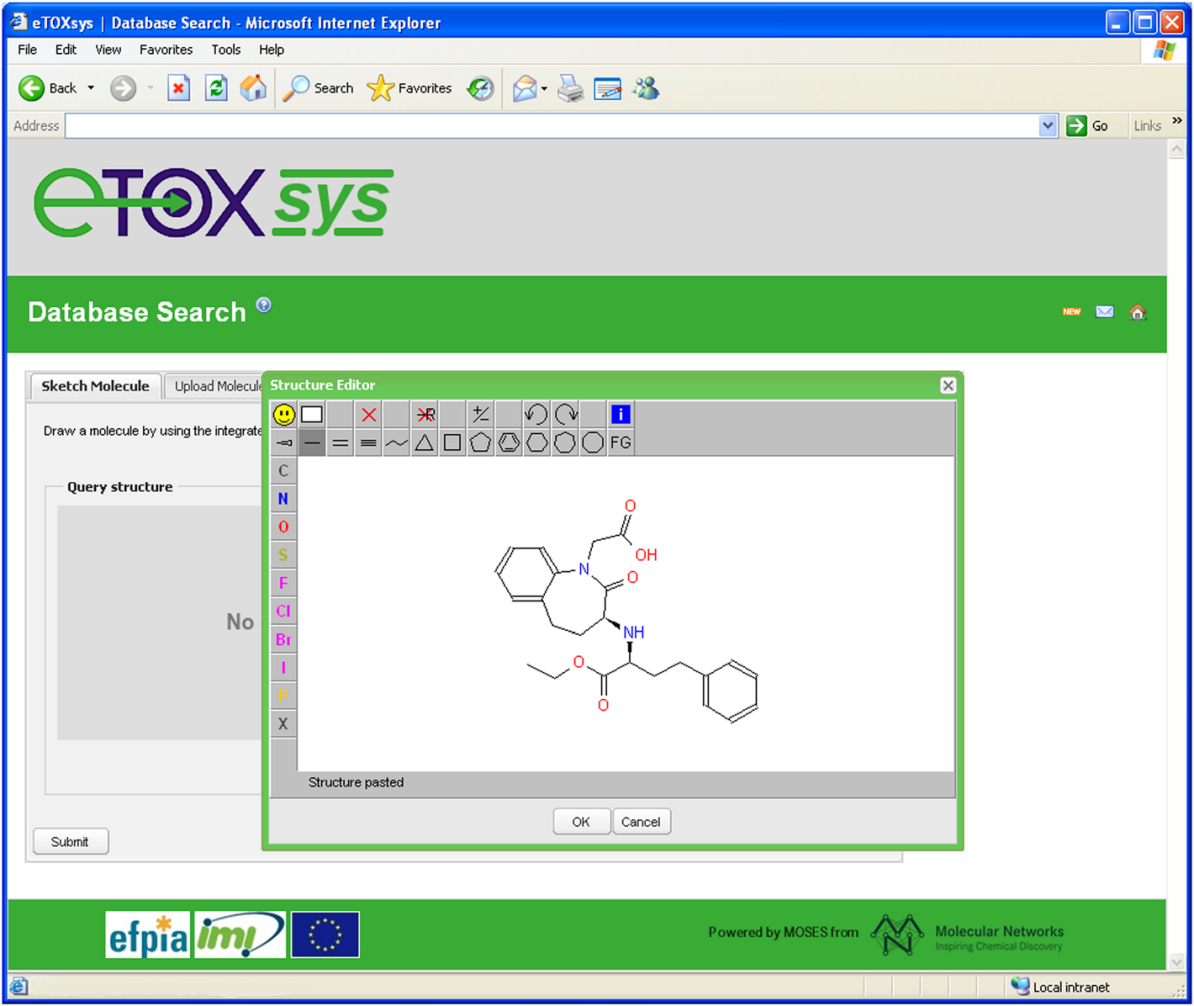
The JSME editor in the “classics” look running in the development version of the eTOXsys system in Internet Explorer 6.

JSME has been tested with success on all major browsers (Internet Explorer, Firefox, Google Chrome, Safari and Opera). Close attention was given to support also the older versions of Internet Explorer (6 and 7) because several large industrial companies still use these legacy versions as their standard browser. Figure 2 shows a screenshot of JSME running within the eTOXsys system [17] in Internet Explorer 6 where it provides a seamless replacement of the JME applet.

In addition to desktop computers running Windows, OS X and Linux operating systems, JSME has been tested also on several touch devices including Apple’s iPhone and iPad and Android smartphones and tablets. Practically all JSME editing features are fully supported (with exception of molecule rotation on Android devices), showing that JSME may be used to edit molecules on many modern smartphones and tablets.

The quality of the graphical display of JSME is browser dependent. On the Internet Explorer versions 6, 7 and 8 (using the VML) when line antialiasing is turned off, the depiction of structures is almost identical to the original Java implementation. Surprisingly, the Google Chrome (tested on version 25 and earlier) provides the worse display quality, where some bonds with a particular angle are not visible when antialiasing is not used. Generally SVG implementation in many browsers seems to have difficulties to render nicely one pixel thick lines without antialiasing. To fine tune the molecule display JSME provides options to change the line thickness of the molecular drawing area and to turn antialiasing on or off.

## Results and discussion

### JSME molecule editor features

**Figure 3:**
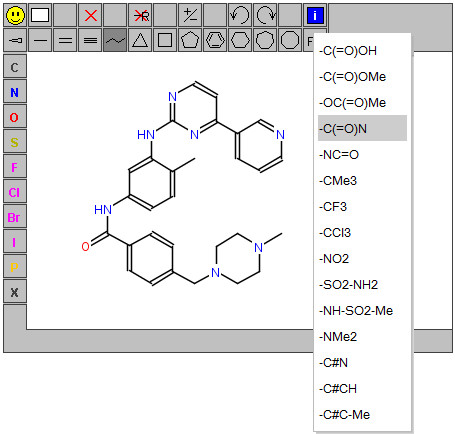
The JSME editor with an open substituent menu.

**Figure 4:**
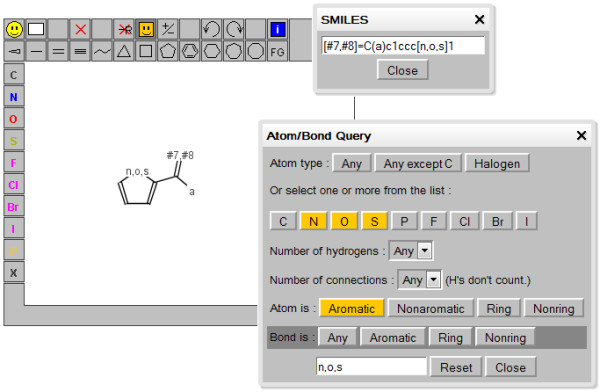
JSME supports generation of complex substructure queries.

**Figure 5:**
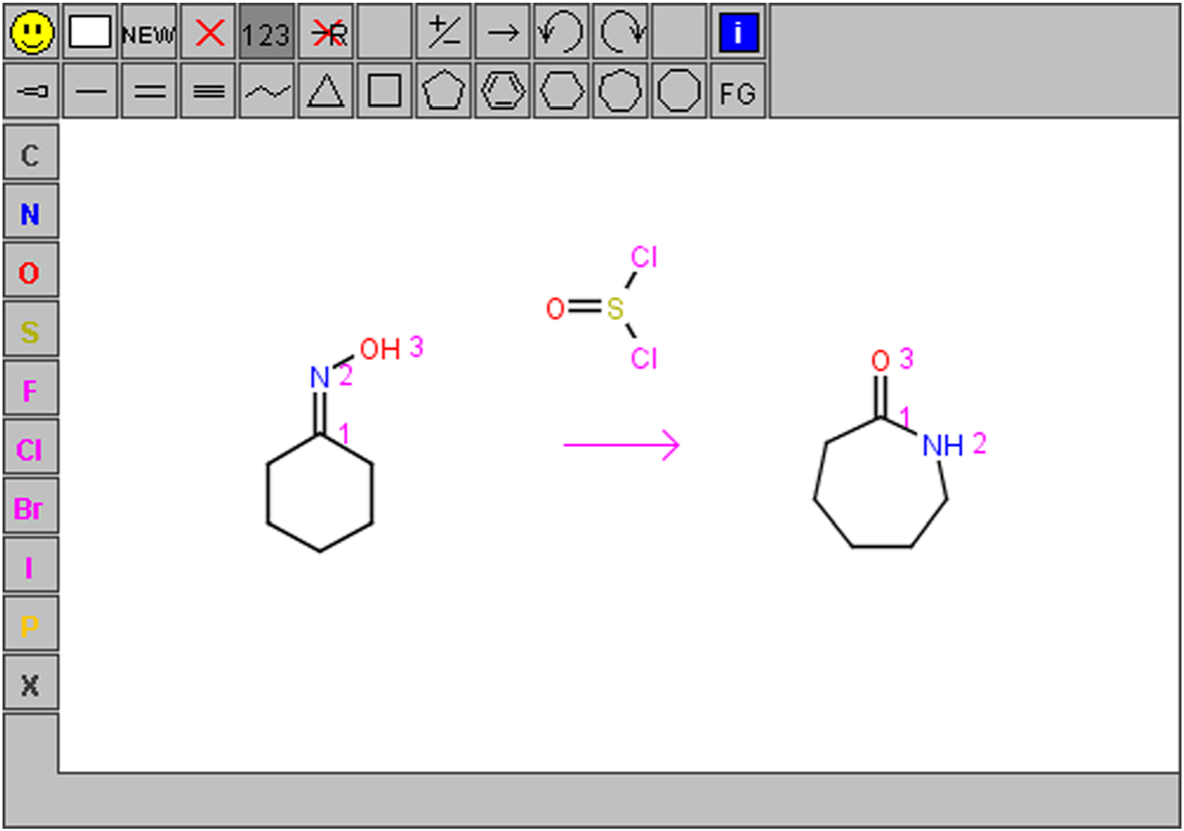
Input of a reaction with atom mapping.

The capabilities of JSME are practically identical to those of JME. JSME supports creation and editing of molecules and reactions (Figure 3). A built-in substituent menu and several keyboard shortcuts provide speedy access to the most common editing features and allow easy and fast creation of even large and complex molecules. The editor is able to export molecules as SMILES [18], MDL/Symyx/Accelrys Molfile [19] or in the compact “JME” format (one line textual representation of a molecule or reaction including also 2D atomic coordinates). The SMILES code generated by JSME is canonical, i.e. independent on the way how the molecule was drawn. The editor can also serve as a query input tool for searching molecular databases by supporting generation of complex substructure queries (Figure 4), which are automatically translated into SMARTS [20]. Input of reactions is also supported (Figure 5), including generation of reaction SMILES and SMIRKS [21]. Molecular structures and reactions can be imported and exported using the system clipboard or the drag and drop feature provided by HTML5 compatible browsers.

Further customization of the editor is possible by executing arbitrary defined JavaScript callback functions when the structure in the editor is modified or when the mouse pointer moves over an atom. It is also possible to change background color of specified atoms and bonds programmatically.

The JSME editor can communicate with other elements on the HTML page via its public functions. These functions are used, among others, to retrieve created molecules, change the editor appearance or programmatically display new molecules. Detailed description of JSME functions is available on-line in the JSME documentation page [22].

To support cheminformatics web community we decided to release the JSME Editor under the terms of permissive BSD license (the license text is included in the distribution package). The editor is available for download from the JSME Home Page http://peter-ertl.com/jsme/ as optimized JavaScript code (the underlying Java code is not provided). We plan to update the JSME distribution on this web page regularly when the new program versions become available.

## Conclusions

We described development and the capabilities of JSME, a free molecule editor written in JavaScript. JSME was developed as direct successor of the popular JME applet, using the JME codebase, therefore although being a new program, it benefits from 15 years of user feedback and bug fixing. JSME is released under the permissive BSD license, which allows the editor to be used freely in both nonprofit and commercial environments. Implementation of JSME in web pages is very simple; therefore we hope that this new molecule editor will become a useful component of numerous new, exciting cheminformatics Internet tools and web services.

## Availability and requirements

The editor is available for download from the JSME Home Page http://peter-ertl.com/jsme/.

## Competing interests

The authors declare that they have no competing interests.

## Authors’ contributions

PE has written the original JME program and modified its Java code to support the translation to JavaScript. BB modified the original Java code, implemented several additional features, did the actual translation to JavaScript and has developed the JSApplet library used in the translation. Both authors wrote the article together. Both authors read and approved the final manuscript.

## Authors’ information

Bruno Bienfait: http://www.molecular-networks.com.

Peter Ertl: http://peter-ertl.com.

## References

[B1] Ertl P Molecular structure input on the Web J Cheminf 2010 2 1 10.1186/1758-2946-2-1 PMC2827360 20298528

[B2] ChemDoodle 2D Sketcherhttp://web.chemdoodle.com/demos/sketcher

[B3] ChemWriter http://metamolecular.com/chemwriter/

[B4] Elemental http://www.dotmatics.com/products/elemental/

[B5] JSDraw http://www.scilligence.com/Web/JSDraw.aspx

[B6] jsMolEditor https://github.com/chemhack/jsmoleditor/

[B7] Ketcher http://ggasoftware.com/opensource/ketcher

[B8] Marvin for JavaScript http://www.chemaxon.com/blog/marvin-for-javascript-released/

[B9] VectorMol http://sciformation.com/vectormol.html

[B10] JSmol http://chemapps.stolaf.edu/jmol/jsmol/test2.htm

[B11] JME Molecule Editorhttp://www.molinspiration.com/jme/

[B12] Ertl P Muehlbacher J Rohde B Selzer P Web-based cheminformatics and molecular property prediction tools supporting drug design and development at Novartis SAR QSAR Env Res 2003 14 321 328 10.1080/10629360310001673917 14758976

[B13] GWT - Google Web Toolkit https://developers.google.com/web-toolkit/

[B14] The Eclipse Foundation http://www.eclipse.org

[B15] Apache Harmony http://harmony.apache.org/

[B16] GWT Mosaic http://code.google.com/p/gwt-mosaic/

[B17] Briggs K Cases M Heard DJ Pastor M Pognan F Sanz F Schwab CH Steger-Hartmann T Sutter A Watson DK Wichard JD Inroads to predict *in vivo* toxicology—an introduction to the eTOX project Int J Mol Sci 2012 13 3820 3846 10.3390/ijms13033820 22489185 PMC3317745

[B18] SMILES - A Simplified Chemical Language http://www.daylight.com/dayhtml/doc/theory/theory.smiles.html

[B19] Chemical Table File http://en.wikipedia.org/wiki/MDL_molfile

[B20] SMARTS - A Language for Describing Molecular Patterns http://www.daylight.com/dayhtml/doc/theory/theory.smarts.html

[B21] SMIRKS - A Reaction Transform Language http://www.daylight.com/dayhtml/doc/theory/theory.smirks.html

[B22] JSME Homepage http://peter-ertl.com/jsme/

